# Impact of Pasta Intake on Body Weight and Body Composition: A Technical Review

**DOI:** 10.3390/nu15122689

**Published:** 2023-06-09

**Authors:** Lisa M. Sanders, Joanne Slavin

**Affiliations:** 1 Cornerstone Nutrition, LLC, Battle Creek, MI 49015, USA; 2 Department of Food Science and Nutrition, University of Minnesota, St. Paul, MN 55108, USA

**Keywords:** pasta, obesity, BMI, dietary patterns, adiposity

## Abstract

Pasta is a staple carbohydrate across many cultures but has been implicated in overweight and obesity due to its position as a refined carbohydrate. Yet, the unique structure of pasta and its low glycemic response suggest it may contribute to a healthy body weight. The purpose of this review is to summarize the literature on the effects of pasta and dietary patterns high in pasta on body weight and body composition outcomes, and evaluate potential mechanisms by which pasta may influence body weight. A search of PubMed and CENTRAL identified 38 relevant studies examining pasta intake and body weight outcomes or potential mechanisms. Observational studies generally report no association or an inverse association of pasta intake with body weight/body composition outcomes. One clinical trial reported no difference in weight loss between a hypocaloric diet with high intake vs. low intake of pasta. Pasta may influence body weight via its low glycemic response, but evidence of effects on appetite, appetite-related hormones, and gastric emptying is limited and inconclusive. In conclusion, observational and limited clinical data suggest pasta is either inversely or not associated with overweight or obesity in healthy children and adults, and does not contribute to weight gain within the context of a healthy diet.

## 1. Introduction

With the growing popularity of low-carbohydrate diets, many traditional carbohydrate foods, such as pasta, have been implicated as contributors to overweight and obesity. Pasta intake in the U.S. and other countries has declined, partially due to the perception of pasta as a “fattening” food [1,2] and also due to declining portion sizes [3,4]. Yet, pasta has a rich culinary history and has been used in dishes around the world for centuries, long before the emergence of the obesity epidemic [5]. Pasta is frequently consumed as part of the Mediterranean diet, which is one of the most well-researched diets for weight loss and reduction of risk of type 2 diabetes (T2D) and cardiovascular disease [6,7,8]. Pasta intake is also associated with higher diet quality; however, this can be impacted by other diet components consumed with pasta in mixed dishes [9].

Pasta is made from refined grain, which may contribute to its perception as “fattening” [2], but unlike many other refined grains, the unique structure of pasta makes it a low-glycemic carbohydrate. Low-glycemic-index (GI)/load diets have been shown to be beneficial for weight loss or maintenance in adults and children [10,11], even when the low-GI diet includes pasta [12,13]. However, the precise role of pasta in obesity and weight management is unclear because most studies examining healthy dietary patterns are unable to determine the role of a single food component such as pasta.

The objective of this study is to summarize the available literature on the role of pasta alone and within healthy dietary patterns on body weight outcomes, such as BMI, weight loss, or odds of overweight/obesity as well as body composition (e.g., abdominal obesity, fat mass, lean mass). Additionally, potential mechanisms by which pasta may influence body weight or body composition will also be reviewed, including appetite, appetite-related hormones, gastric emptying, and glycemic response.

## 2. Materials and Methods

A literature search was conducted using PubMed and CENTRAL databases to identify observational studies and clinical trials examining pasta intake and body weight outcomes. Searches also included terms for dietary patterns where pasta is commonly consumed (e.g., Mediterranean diet, low-GI diet) and potential mechanisms by which pasta may contribute to weight management, such as appetite. The search was limited to English language publications. Full search terms are provided in the Supplemental Materials (Table S1).

Inclusion criteria comprised prospective cohort, cross-sectional, and clinical trials in healthy adults and children. Interventions included diets or meals containing pasta compared to similar diets or meals with less or no pasta. Pasta made from durum wheat was included as this is the most common ingredient used to make pasta globally and its compact structure may influence physiological effects. In observational studies, the primary exposure variable was pasta consumption or diets higher in pasta compared to lower pasta consumption or diets lower in pasta. Higher and lower amounts of pasta were not pre-specified but based on the definition of “higher” and “lower” pasta intake reported in each individual study. Observational studies on dietary patterns that did not include assessments of differences in pasta intake were excluded. Since observational studies do not define the type of pasta consumed or ingredients used, these studies could not be restricted to durum wheat pasta. Retrospective, case-control, and single-arm (no-control) studies were excluded, as were studies conducted in vitro, or in animals. Studies were also required to include a body weight or body composition outcome (e.g., BMI, weight loss, percent body fat, etc.) or a physiological outcome related to weight management (e.g., subjective appetite, appetite-related hormones, gastric emptying, etc.). Exclusion criteria consisted of studies in pregnant or lactating women, and studies in individuals with a chronic disease or condition, except for overweight/obesity. Trials with noodles (e.g., rice noodles, egg noodles), gluten-free pastas, or pastas not made from durum wheat were not included. Trials that utilized pastas fortified with additional components (e.g., high-protein pasta, fiber-fortified pasta) or whole-grain pasta only were also excluded since these pastas are either not available on the market or constitute a small amount of pasta sold globally [14]. After removal of duplicates, screening of titles and abstracts was conducted to determine potential eligibility. Full texts of potentially eligible publications were obtained and reviewed for inclusion. Reference lists were also examined for additional publications to include.

## 3. Results

Following the search and full-text review, 38 publications were identified for inclusion. A total of 12 of these publications were observational studies examining the relationship of pasta intake to body weight or body composition outcomes [4,15,16,17,18,19,20,21,22,23,24,25]. An additional 15 publications were observational studies evaluating the impact of dietary patterns with varying amounts of pasta on body weight or body composition outcomes [26,27,28,29,30,31,32,33,34,35,36,37,38,39,40]. One clinical trial was identified that evaluated the effect of varying pasta intake on body weight outcomes [41]. A total of 10 clinical trials evaluated the impact of pasta on potential mechanisms related to body weight and body composition, including appetite [42,43,44,45,46], appetite-related hormones [44,47], food/energy intake [43,44,45,46], glycemic response [44,45,47,48,49,50,51], and gastric emptying [47].

### 3.1. Observational Studies on Pasta Intake and Body Weight Outcomes

Six publications reported an inverse association of pasta intake with one or more body weight outcomes, including BMI [17,19,20,23], waist circumference (WC) [19], hip circumference (HC) [20], waist-to-hip ratio (WHR) [21], and odds of overweight/obesity [15]. Pounis et al. [20] and Papanikolaou [19] reported these associations in females but not males, and Shay et al. [23] in males but not females. Six publications reported no association of pasta intake with one or more body weight outcomes [4,18,19,20,22,24]. Three studies reported a positive association of pasta intake and body weight change, BMI, or HC. Palmer et al. [16] interviewed university students in Germany during the COVID-19 lockdown in 2020 and found self-reported increases in pasta intake (based on a seven-point Likert scale from less to more) were associated with self-reported increases in body weight during lockdown. Kelishadi et al. [25] reported that higher pasta intake compared to lower pasta intake in Iranian adolescents was associated with higher BMI. Adolescents with overweight/obesity also reported lower physical activity and greater time spent watching television. Krachler et al. [24] reported a positive association of pasta intake and HC in men only and no association with WC.

Studies were conducted around the globe, but most were in European countries, such as Spain, Italy, Sweden, and Norway. Study outcomes did not appear to differ by country. Most studies included adults, but two studies also included children [4,19] and four were exclusively in children or adolescents [17,18,22,25]. One study exclusively in children across six European countries reported an inverse association of pasta intake and BMI [17], but another study in Iranian adolescents reported a positive association of pasta intake and BMI [25]. The remaining publications reported no association of pasta intake with BMI, WC, or odds of overweight/obesity in children and adolescents, although one publication did not include a separate analysis for children and adults [4]. While most studies evaluated the relationship to pasta alone, three studies combined pasta with intake of other starchy foods, such as rice and/or potatoes [15,21,22]. Two of these studies reported an inverse association of pasta/rice intake and pasta/rice/potato intake with WHR and odds of overweight/obesity, respectively [15,21]. Another study reported no association of pasta/rice intake with odds of overweight/obesity [22]. Table 1 provides a summary of observational studies assessing pasta intake and body weight/composition.

While most observational studies utilized food frequency questionnaires to estimate pasta intake and did not collect information on specific amounts consumed or the nutritional composition (e.g., fiber, whole-grain content) of the pasta, some studies were able to give an estimate of pasta intake. Shay et al. [23] reported that median pasta intake for men in the high BMI group (≥30) was 21.5 g/1000 kcals compared to 36.4 g/1000 kcals in the lowest BMI group (<25). Periera et al. [4] and Grosso et al. [22] reported no significant change in the OR for overweight/obesity for every 10 g pasta consumed. Papanikolaou [19] analyzed nutrient intake and determined adult and child pasta consumers compared to pasta non-consumers had higher dietary fiber and lower saturated fat intake across the day, but the specific contribution of pasta is unclear.

### 3.2. Observational Studies of Dietary Patterns Higher in Pasta Compared to Dietary Patterns Lower in Pasta and Body Weight/Composition Outcomes

**Figure 1:**
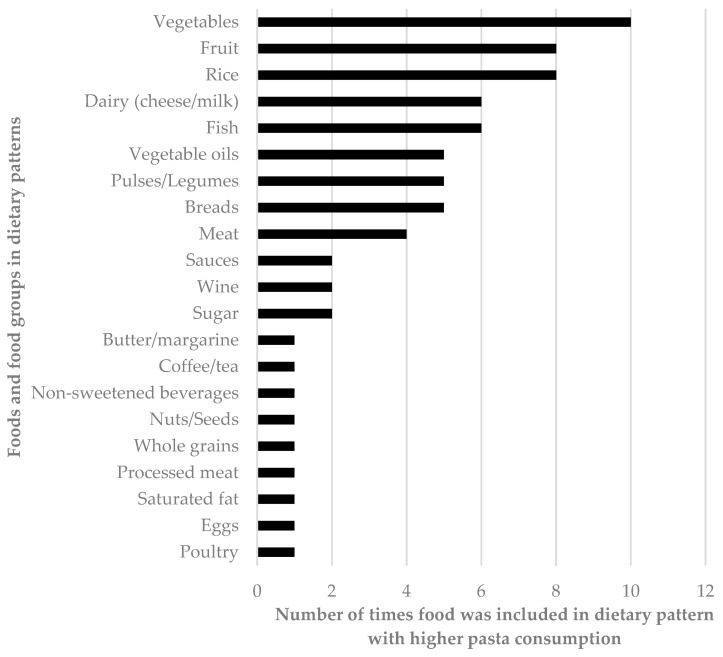
Additional food groups characterizing dietary patterns high in pasta.

Dietary patterns reported in the literature were very diverse, although most dietary patterns higher in pasta were also higher in vegetables, fruits, rice, dairy, and fish than other dietary patterns (Figure 1). Seven studies reported an inverse relationship of dietary patterns high in pasta with body weight or body composition outcomes [28,30,32,33,36,39,40]; however, four of these studies included mixed results for different outcomes [33,36,39,40] (Table 2). Five studies reported no associations of dietary patterns high in pasta with body weight or body composition [26,27,29,31,37], while three reported only positive associations [34,35,38]. Studies assessing BMI (or BMI z-score for children) either reported no association of dietary patterns high in pasta with BMI [29,31,36,40] or a positive association [38,39]. Most of the studies reporting no association were in children [29,31,36], with one being in middle-aged adults [40], while both studies reporting a positive association were in older (≥60 years) Europeans from the European Prospective Investigation into Cancer and Nutrition (EPIC) cohort [38,39]. Both of these EPIC studies also reported that greater adherence to a diet higher in sweets was associated with lower BMI, particularly in women. A study in Spain reported college students with greater compliance to the Mediterranean diet (which included pasta consumption) were more likely to have overweight or obesity (BMI > 25) [34]. However, this did not seem to be driven by the pasta component of the diet since pasta intake was similar in individuals with normal weight and overweight.

Dietary patterns high in pasta were generally not associated with odds of overweight or obesity [27,37] or were inversely associated [28,30]. One study in Mexican adults reported greater odds of overweight or obesity in individuals consuming a diverse dietary pattern (whole-fat dairy, rice and pasta, meat, poultry, eggs, saturated fat, fruits, and vegetables) compared to a traditional diet (low dietary diversity, maize and maize foods ~50% of energy intake, beans), despite significantly lower energy intake and improved nutrient intake with the diverse diet [35]. Ng et al. [26] also found pasta and rice to not be predictive of an obesogenic diet in Canadian adults.

Dietary patterns higher in pasta intake compared to dietary patterns lower in pasta intake were more consistently inversely associated with abdominal obesity. Four studies reported an inverse relationship of dietary patterns high in pasta with WHR [32,39,40] and odds of abdominal obesity [30]. However, one study reported a positive association with WHR in elderly Italians consuming a pasta-and-meat dietary pattern [38]. Voortman et al. [32] examined dietary patterns using three different methods and found an inverse relationship of pasta intake at one year of age to abdominal obesity at age seven with one method (reduced rank regression), but no association using two other methods (diet quality score, principal component analysis).

Few studies assessed body fat or lean mass composition, and results were mostly mixed. Voortman et al. [32] examined dietary patterns at one year of age and reported no association of dietary patterns higher in pasta with fat mass at age seven, but a positive association of diets lower in pasta with fat mass. Further, dietary patterns higher in pasta were positively associated with fat-free mass at age seven. Similarly, Smith et al. [33] reported a decrease in fat mass gain in 9–11 years girls consuming a dietary pattern higher in pasta compared to a dietary pattern lower in pasta, but a similar effect was not observed in boys. Interestingly, a dietary pattern higher in pasta was also associated with less gain in lean mass in girls but not in boys. In a study of weaning diets and body composition at four years of age, Robinson et al. [36] reported a positive association of weaning diets based on recommendations (fruit, vegetables, meat and fish, and other home-prepared foods such as rice and pasta) with lean mass.

### 3.3. Clinical Trials on Pasta Intake and Body Weight/Composition Outcomes

One quasi-experimental trial in Italy examined the impact of pasta intake on body weight outcomes [41]. In this trial participants (*n* = 49) were instructed to consume high (five or more 80 g svgs/wk) or low (no more than three 80 g svg/wk) amounts of pasta as part of a six-month Mediterranean-style hypocaloric diet [41]. Nutrient intake, including dietary fiber, did not differ between the groups over the six-month intervention. Both groups lost weight and experienced reduced WC, HC, and fat mass, but there were no differences in anthropometric outcomes between the high- and low-pasta-intake groups. There were no additional studies comparing dietary patterns (e.g., Mediterranean, low-GI) with pasta to the same dietary pattern without pasta.

### 3.4. Clinical Trials on Potential Mechanisms by Which Pasta May Influence Body Weight and Body Composition

The lower glycemic response of pasta compared to other starchy foods was the most consistent outcome in clinical trials (six of seven studies, 86%). Most trials reported a lower glycemic response of pasta compared to a similar amount of carbohydrate from bread [47,48,50], white rice [47,49,51], mashed potatoes [47], and Asian noodles [51]. Akilen et al. [44] reported a higher glycemic response for pasta compared to French-fried potatoes, but no difference from rice, boiled and mashed potatoes, and baked French fries. This may be due to cooking conditions, as the French-fried potatoes contained five times the fat of pasta. The potential influence of cooking conditions was also demonstrated in one study that reported baked potatoes had a similar glycemic response as pasta, while instant mashed potatoes had a higher glycemic response [45]. Additionally, when brown rice was used instead of white rice, the glycemic response was similar to that of pasta [45]. Asian noodles differed from pasta in the type of wheat used as well as the process of making the noodles, which may explain differences in the glycemic response [51,52]. Dietary fiber content of the test meals was not always reported, but for those that did, the dietary fiber content of pasta was similar to white bread and potato (0–3.7 g/serving), but greater than rice (0–1.6 g/serving) and Asian noodles (1.3 g/serving).

Pasta consumption generally resulted in similar subjective satiety and hunger compared to other starchy foods, such as rice and potatoes [42,43,44,45,46]. Erdmann et al. [46] demonstrated that satiety persisted for longer after a meal containing pasta compared to a meal with potato; however, the participants were allowed to consume ad libitum amounts of potato and pasta, and energy intake was less with the potato meal compared to the pasta meal. Thus, it is unclear if the persistence of satiety was due to the pasta or due to the higher energy intake in the pasta meal. Diaz-Toledo et al. [43] reported greater satiety with French-fried potatoes compared to pasta, but pasta did not differ from other preparations of potatoes (mashed, baked, wedges). Studies evaluating appetite-related hormones reported similar effects among starchy foods on glucagon-like peptide-1 (GLP-1) and peptide YY (PYY), but a lower ghrelin response with pasta compared to potatoes and rice [44,46]. However, both of these studies were designed for ad libitum consumption of the starchy food, so the amounts of pasta, potato, or rice were not the same (by grams of carbohydrate, volume, or energy).

Studies assessing food and energy intake, a critical component of weight management, reported no difference in energy intake at a subsequent meal after consuming a test meal with pasta or potatoes [43,44,45]. However, energy intake within a meal differed based on the starch component. When participants were asked to eat a meal until satiated, food intake was similar when the starch served was pasta, rice, or potato, but overall energy intake was lower with potatoes compared pasta or rice, likely due to the lower energy density of potatoes [44,46]. The rate of gastric emptying can also influence food intake and glycemic response, and Mourot et al. [47] reported the gastric emptying rate of pasta was slower than other starchy foods, including bread, rice, and mashed potatoes.

## 4. Discussion

Overall, results from most observational studies suggest pasta and dietary patterns higher in pasta intake were either inversely associated or not associated with body weight and body composition outcomes, such as BMI, odds of overweight/obesity, or abdominal obesity. Only one clinical trial was identified examining the impact of pasta intake alone on weight loss, and was consistent with observational studies suggesting that pasta can be included in a healthy diet and not contribute to weight gain or hinder weight loss [39].

The results from observational studies were generally consistent across different populations, including adults and children, as well as in different regions of the world. This is important since pasta composition can vary around the world, with most European countries, particularly Italy, using 100% durum wheat, while other countries may allow pastas with blends of durum and other wheat flours [5,53]. When pasta was classified and evaluated in combination with other starchy foods, such as rice, potatoes, and bread, there remained either an inverse association with WHR and odds of overweight/obesity [15,22] or no association with odds of overweight/obesity [21]. However, as demonstrated in clinical trials, the effects of these starchy foods on outcomes that may influence body weight, such as glycemic response and gastric emptying, can vary considerably. Thus, it may be preferable to differentiate between these starches in observational studies to better elucidate the specific effects of pasta on body weight and body composition outcomes.

The majority of observational studies reported either an inverse or no association of pasta intake and body weight or body composition outcomes, although the associations may not be strong considering the small beta coefficients and odds ratios. Some studies also reported positive associations of pasta intake or dietary patterns higher in pasta and body weight outcomes [24,35,38]. However, this may be due to other dietary factors characteristic of the dietary pattern or potential confounding with non-dietary factors. While most studies included pasta in a dietary pattern also higher in vegetables, legumes, fruits, and fish, compared to other dietary patterns, this was not the case for all dietary patterns investigated. For example, in Pala et al. [38], the dietary pattern high in pasta was also characterized by higher intake of red meats and processed meats, which have been associated with increased risk of overweight and obesity [54,55]. Similarly, a study in Mexican adults reported a dietary pattern higher in pasta was also characterized by higher saturated fat intake [35]. It is possible other components within the dietary pattern may contribute to the associations observed with body weight outcomes. Pasta is often included in mixed dishes which can contain a variety of foods including vegetables, vegetable sauces, seafood, and poultry, as well as foods higher in saturated fat, such as red and processed meats and cheese. Fulgoni et al. [9], using national representative U.S. data, reported higher diet quality in pasta consumers compared to non-consumers, but lower diet quality if the pasta dish consumed was macaroni and cheese, indicating the importance of other components consumed along with pasta.

Two studies from the elderly population of the EPIC cohort reported positive associations of dietary patterns higher in pasta with BMI, but also reported a similar positive association with a prudent diet [38] and an inverse association of BMI with a “sweets and dairy” diet [39]. These results suggest there may be non-dietary confounders partially responsible for the relationship between dietary patterns and body weight or body composition. For example, Pala et al. [38] reported physical activity to be lowest in female consumers of the prudent diet, which may have contributed to the positive association of this diet with BMI. To help control for these potential confounders, as well as the influence of other dietary components, randomized controlled trials are needed that provide similar dietary patterns only varying in pasta intake while also minimizing or eliminating potential non-dietary differences.

The study by Rosi et al. [41] is an example of the type of clinical trial needed to understand the role of pasta on body weight outcomes. While the study demonstrated that similar weight loss was achievable with high (80 g svg, five times per week) and low (80 g svg, less than three times per week) intake of pasta within a hypocaloric diet, the design was quasi-experimental and more studies with a randomized controlled design are needed. It is also important to consider that there have been many clinical trials on low-GI or Mediterranean diets, both commonly characterized by higher pasta intake. In a systematic review and meta-analysis by Chiavaroli et al. [12], the researchers reported the inclusion of pasta in a low-GI diet did not contribute to weight gain, and weight loss was greater compared to high-GI patterns containing less pasta. Similarly, a review from Esposito et al. [7] reported greater weight loss with a Mediterranean diet and no evidence of increases in body weight; however, the review did not evaluate pasta intake specifically, and pasta intake varies across the Mediterranean region [8]. Continued studies on these dietary patterns are important, but to better discern the true effect of pasta on body weight and body composition within a healthy diet, randomized controlled trials of these healthy dietary patterns varying only in pasta intake are needed.

The potential mechanisms by which pasta may contribute to a healthy body weight or body composition are less clear, but the most consistent evidence suggests the low glycemic response of pasta may play a role. Within this review and others [56], clinical trials consistently demonstrate pasta to have a lower glycemic response compared to similar starchy foods, such as rice, potatoes, bread, and Asian noodles [45,47,48,49,50,51]. There may be advantages to body weight when the diet is composed of low-glycemic carbohydrates instead of high glycemic carbohydrates [57,58]. Increases in blood glucose levels, which are greater with high glycemic carbohydrates, trigger the release of insulin, which acts to clear glucose from the bloodstream. Insulin has also been shown to increase hunger, which may trigger food consumption, an increase in food intake, and possible weight gain over time [57]. However, well-controlled clinical trials evaluating the impact of glycemic load on body weight and body composition, within an energy-controlled diet, are still needed.

Numerous factors may contribute to the lower glycemic response of pasta, including the compact food structure, slowly digestible starch and fiber content, proportion of durum wheat vs. other wheat flours, and slower gastric emptying [47,50]. The process of making pasta results in a highly compact structure with cross-linking of gluten proteins which encapsulates the starch, limiting its accessibility to digestive enzymes [50,59]. Additionally, the cooking and subsequent cooling of pasta can result in starch retrogradation, leading to resistant starch formation, which may slow digestion [60]. This also happens during the baking and cooling of potatoes, which may partially explain why the glycemic response of pasta and baked potato were not significantly different in one trial [45]. As a result of these structural components, most starch in pasta is slowly digested as evidenced by large amounts of nondigested starch embedded in the pasta protein matrix after mastication and in vitro digestion, while the starch in bread is almost completely digested [50]. Dietary fiber content may also impact the glycemic response [61], although this depends on the type and amount of dietary fiber consumed [62]. While not all studies in the current review reported dietary fiber content in the test meals, those that did reported relatively small differences in fiber content between pasta and other starchy foods (≤3 g/serving). This suggests it is more likely that differences in the food structure of pasta, rather than the fiber content, are responsible for differences in glycemic response.

It may be expected that a slower digestion and lower glycemic response of pasta would lead to differences in appetite; however, the evidence is inconclusive due to limited data and highly variable study designs. As an example, the studies on subjective appetite did not always match carbohydrate amounts or volume or energy intake in the test meals, each of which can impact appetite. Zhang et al. [42] reported greater hunger after consuming pasta compared to potato, but the volume of potato provided (337 g) was more than double that of pasta (138 g) since the meals were matched to contain 45 g carbohydrate. Pasta, due to its compact structure, has more carbohydrates per gram than potato. Other studies measured satiety after ad libitum consumption of starchy foods, which makes it difficult to determine if the response is due to the pasta or to the energy intake or volume of food consumed [44,46]. One trial attempted to match available carbohydrate, fiber (0–3 g/serving), volume, and energy intake in the test meal and reported no differences in subjective hunger or fullness between pasta, rice, or potato meals and no difference in energy intake at a subsequent meal [45].

The strengths of this review include a comprehensive search over two databases, inclusion of observational and clinical trial data in adults and children, and examination of potential mechanisms in addition to observed effects on body weight and body composition. Additionally, the focus on healthy individuals makes this review relevant to the general population. However, there are important limitations to note. Most of the data on pasta intake and body weight outcomes are from observational studies which do not determine causality and are subject to confounding. While most studies used covariates such as age, sex, physical activity, and/or energy intake, these adjustments cannot account for all potential confounding. Observational studies also rely on self-reported food intake, and several of the studies in the current review also used self-reported body weights, which may not be an accurate reflection of intake or body weight. Additionally, cross-sectional studies only assess intake and body weight at a single time point but cannot determine changes over time. While there is one clinical trial examining the causal relationship of pasta and weight loss, it was also limited by its lack of randomization and small sample size after dropouts, so more randomized controlled trials are needed before causality can be determined. An additional limitation is a lack of description of the pasta, particularly in observational studies. Pastas can vary considerably around the globe as to the % durum wheat included [5,53]. While we excluded studies in noodles, which are generally made from soft wheat flours, it is unclear what portion of the pasta in observational studies is made from durum wheat. This review also did not include whole-grain pasta or pasta with additional functional ingredients, such as fiber. Because of its versatility, numerous studies have evaluated the addition of different grains, legumes, seeds, vegetables, and fiber ingredients to create functional pastas, which may enhance potential benefits for body weight.

## 5. Conclusions

In conclusion, despite the perception of some that pasta is a “fattening” food [2], current observational evidence suggests pasta is generally not associated with body weight or body composition and may be inversely associated with BMI or abdominal obesity, particularly when consumed in the context of a healthy dietary pattern. While additional randomized controlled trials are needed to confirm these observational findings, one recent study demonstrated that pasta does not hinder weight loss or contribute to weight gain within a healthy dietary pattern [41]. The mechanisms by which pasta may influence body weight are less clear, but slow digestibility resulting in a lower glycemic response may play a role and should be investigated further. Dietitians and other health care professionals should consider pasta as a low-glycemic carbohydrate option within the context of a healthy diet or a weight loss/weight maintenance diet.

## Figures and Tables

**Table 1:** Summary of observational studies assessing pasta intake and body weight/composition outcomes.

Reference	Design	Population	Dataset and Location	Outcomes	Key Findings
De Pedro-Jiminez et al. (2022) [15]	Cross-sectional	9097 adults (18–65 years)	Spain Spanish National Health Survey 2017	Odds of ow/ob	OR ow/ob 1.2 (1.03–1.4) for low (<1 time/week) consumption. OR ow/ob 1.16 (1–1.34) for moderate (1–3 times/week) consumption of pasta/potatoes/rice compared to high consumption.
Palmer et al. (2021) [16]	Cross-sectional	827 university students	Germany During 2020 COVID-19 lockdown	BMI	Self-reported increase in pasta intake positively associated with self-reported increase in BMI (β = 0.097, *p* < 0.005).
Hormann-Wallner et al. (2021) [17]	Cross-sectional	330 children (9–12 years)	Six European countries	BMI	Inverse association of BMI for age and pasta intake (r = −0.127, *p* = 0.021).
Androutsos et al. (2021) [18]	Cross-sectional	397 children (2–18 years)	Greece	Change in body weight	No association of pasta intake with parent-reported changes in body weight.
Papanikolaou (2020) [19]	Cross-sectional	400 adults 323 children (2–18 years)	United States NHANES 2001–2012	Body weight BMI WC Skinfolds	No association across quartiles of pasta intake with body weight, BMI, WC, or skinfolds in adults or children. Subgroup analysis of women 19–50 years demonstrated inverse association across quartiles of pasta intake with body weight, BMI, and WC.
Pereira et al. (2018) [4]	Cross-sectional	5270 individuals (≥12 years)	Brazil Health Survey of Sao Paulo 2003–2015	Odds of ow/ob	Portion sizes of pasta have decreased from 2003 (mean = 238.5 g) to 2015 (mean = 192.1 g). No association of pasta portion size with odds of ow/ob.
Pounis et al. (2016) [20]	Cross-sectional	14,402 adults	Italy Moli-sani cohort and Italian Nutrition and Health Survey (INHES)	BMI WC HC WHR	Moli-sani cohort Inverse association of pasta intake with BMI and HC in women, but not men (women β_BMI_ = −0.007, *p* = 0.003; women β_HC_ = −0.01, *p* = 0.03). No association of pasta intake with WC or WHR. INHES No association of pasta intake and self-reported BMI (did not assess WC, HC, or WHR).
Mostad et al. (2014) [21]	Cross-sectional	541,093 adults	Norway HUNT3 survey 2006–2008	WHR	Inverse association of pasta/rice intake frequency and WHR.
Grosso et al. (2013) [22]	Cross-sectional	1135 adolescents (13–16 years)	Spain	Odds of ow/ob	Positive association of pasta/rice intake with Mediterranean diet adherence (β = 0.019, *p* < 0.001). No association of pasta/rice intake with OR of ow/ob.
Shay et al. (2012) [23]	Cross-sectional	2195 adults (40–59 years)	United States INTERMAP	BMI	Inverse association of BMI and pasta/rice intake in men only (β = −0.64, *p* < 0.01).
Krachler et al. (2006) [24]	Cross-sectional	5915 adults	Sweden MONICA cohort	WC HC	Positive association of pasta intake with HC in men (+1.59 mm, *p* = 0.02). No association of pasta intake with WC in both genders or HC in women.
Kelishadi et al. (2003) [25]	Cross-sectional	2000 adolescents (11–18 years)	Iran	BMI	Positive association of pasta intake and BMI (β = 0.03, *p* = 0.04).

Abbreviations: HC = hip circumference; WC = waist circumference; WHR = waist-to-hip ratio; ow/ob = overweight/obesity.

**Table 2:** Summary of body weight/composition outcomes in observational studies on dietary patterns differing in pasta intake.

Outcome	Reported Outcomes with Inverse Association	Reported Outcomes with No Association	Reported Outcomes with Positive Association
BMI or BMI z-score	0	4	2
OR overweight/obesity	2	2	1
Abdominal obesity (WC, WHR, or OR)	4	1	1
Fat mass, % body fat	2	1	0
Fat-free mass	0	0	1
Lean mass	1	0	1

Abbreviations: OR = odds ratio, WC = waist circumference, WHR = waist-to-hip ratio. Green shading indicates the association is desirable. Red shading indicates the association is not desirable.

## Data Availability

No new data were created or analyzed in this study. Data sharing is not applicable to this article.

## Supplementary Materials

The following supporting information can be downloaded at: https://www.mdpi.com/article/10.3390/nu15122689/s1, Table S1: Search terms.
