# Supplementary material for: Impact of Pasta Intake on Body Weight and Body Composition: A Technical Review
Source: Nutrients. 2023 Jun 9;15(12):2689. doi: 10.3390/nu15122689 (PMC10300797; doi:10.3390/nu15122689)
Supplement: Supplementary file 1 [file nutrients-15-02689-s001.zip › nutrients-2414181-supplementary.pdf]

Impact of pasta intake on body weight and body composition: A technical review

Supplemental Materials

Lisa M. Sanders and Joanne Slavin

Table S1: Search terms

**Pasta Search Terms:** "pasta" OR "capellini" OR "farfalle" OR "fettuccine" OR "lasagna" OR "macaroni" OR "manicotti" OR "orecchiette" OR "orzo" OR "penne" OR "rotini" OR "tortellini" OR "fusilli" OR "noodle"
**Obesity Search Terms:** "body weight" OR BMI OR "body mass index" OR "waist circumference" OR "BMI z-score" OR "weight z-score" OR "weight loss" OR "weight gain" OR "weight change" OR "weight maintenance" OR obesity OR overweight OR "body fat" OR "adiposity" OR "waist-to-hip ratio"
**Dietary Patterns Search Terms:** "dietary pattern" OR "Mediterranean diet" OR "vegetarian diet" OR "vegan diet" OR "plant-based diet" OR "Low glycemic index diet" OR "Low GI diet" OR "dietary approaches to stop hypertension" OR "DASH diet" OR "portfolio diet" OR "Healthy US-Style" OR "high carbohydrate diet" OR "low carbohydrate diet"
**Mechanisms Search Terms:** "appetite" OR "satiety" OR "hunger" OR "fullness" OR "desire to eat" OR "prospective food consumption" OR "dietary intake" OR "food intake" OR "appetite hormone" OR "gut hormone" OR "amylin" OR "cholecystokinin" OR "CCK" OR "corticotropin-releasing" OR "dopamine" OR "ghrelin" OR "glucose-dependent insulinotropic polypeptide" OR "GIP" OR "glucagon-like peptide 1" OR "GLP-1" OR "glucagon" OR "insulin" OR "leptin" OR "oxyntomodulin" OR "pancreatic polypeptide" OR "peptide YY" OR "PYY" OR "serotonin" OR "colonic transit time" OR "gastric emptying time" OR "dietary fiber" OR "resistant starch" OR "slowly digestible starch" OR "slowly digestible carbohydrate"
